# Social contact patterns and associated factors survey of Shangrao City

**DOI:** 10.3389/fpubh.2026.1866091

**Published:** 2026-07-14

**Authors:** Juan Liu, Wenli Li, Hongfeng Zhao, Yufang Zhao, Xiaomin Zhan, Yanhua Su, Yao Liu, Peng Xu

**Affiliations:** 1 Department of Infectious Disease Control, Shangrao Center for Disease Control and Prevention, Shangrao, Jiangxi, China; 2 School of Public Health, Xiamen University, Xiamen, Fujian, China; 3 Jiangxi Medical College, Shangrao, Jiangxi, China; 4 Department of Infectious Disease Control, Wuyuan County Center for Disease Control and Prevention, Shangrao, Jiangxi, China

**Keywords:** age heterogeneity, cross-sectional survey, infectious disease transmission, respiratory infectious diseases, social contact patterns

## Abstract

**Objective:**

This study aimed to quantitatively analyze social contact patterns among residents of Shangrao City, Jiangxi Province, and to examine their variations across different demographic groups. The findings are intended to provide empirical data for developing localized transmission dynamic models of respiratory infectious diseases and for formulating targeted prevention and control strategies.

**Methods:**

A cross-sectional online survey was conducted among Shangrao residents from April to September 2025, and 2,101 valid questionnaires were analyzed. Daily total, household, and non-household contacts were described by demographic groups. Factors associated with daily total contacts were examined using negative binomial regression, with results reported as incidence rate ratios (IRRs) and 95% confidence intervals. Post-stratification weighting based on the 2020 Seventh National Population Census of Shangrao was performed as a sensitivity analysis.

**Results:**

The mean daily number of contacts was 7.91, including 2.73 household and 5.19 non-household contacts. Adolescents aged 5–19 years had the highest mean daily total contacts (9.99). In the negative binomial regression, the 5–19 years group had more contacts than the 20–29 years reference group (IRR 1.74, *p* < 0.001), and participants with a mobility range >10 km also had more contacts than those with a range ≤1 km (IRR 1.18, *p* < 0.001). These age and mobility effects remained stable after post-stratification weighting, whereas the sex and county/district contrasts were less consistent and should be interpreted cautiously.

**Conclusion:**

Social contact patterns in Shangrao City are dominated by age, with adolescents (5–19 years) and highly mobile populations exhibiting the highest contact frequencies. These conclusions were robust to post-stratification weighting against the 2020 Seventh National Population Census of Shangrao. The study addresses the absence of empirical social contact data in a Chinese prefecture-level city and provides directly applicable evidence for localized epidemic risk assessment and early-warning modelling of respiratory infectious diseases.

## 1 Introduction

Respiratory pathogens are primarily transmitted through droplets generated by infected individuals through behaviors such as coughing and sneezing (1). Social contact serves as the physical medium for the cross-host transmission of pathogens. Under certain spatial distances and contact durations, social contact can facilitate the transmission of respiratory droplets. Therefore, the intensity of social contact is a key factor determining the transmission dynamics of respiratory infectious diseases (2). Higher contact frequencies among individuals or within groups are associated with an increased risk of pathogen transmission and subsequent infection (3).

Contact patterns are also significantly influenced by geographic environment, occupation, and household size. Studies have demonstrated marked differences in contact intensities within households, schools, and workplaces between high- and low-income regions, as well as between urban and rural populations (2, 4, 5). Given the heterogeneity in socioeconomic structures and population densities across regions, existing contact data are often not generalizable at either the global or national level. Empirical contact data from specific regions are therefore essential for developing accurate localized transmission dynamics models.

To date, studies on social contact patterns have been conducted in cities such as Shanghai, Wuhan, and Suzhou, however, empirical data for Shangrao City remain scarce (6). This study aims to quantitatively investigate contact patterns among 2,101 respondents in Shangrao and to analyze variations across demographic groups, thereby informing evidence-based prevention strategies and public health decision-making.

## 2 Materials and methods

### 2.1 Participants

The target sample size was determined by reference to previous social contact pattern surveys in China and by considering the need for broad geographic coverage and subgroup analyses. A recent post-COVID-19 social contact survey in Suzhou, China used a stratified sampling design across eight administrative districts and eight age groups and surveyed 1,608 participants (4). The Shanghai social contact survey also predefined a target sample size of 1,000 participants (7). Based on these comparable studies and the feasibility of field recruitment across the 12 county-level areas of Shangrao City, we aimed to recruit approximately 2,000 residents. Ultimately, 2,119 individuals participated in the survey, and 2,101 valid questionnaires were retained for analysis.

The survey was conducted among residents of the 12 county-level areas within Shangrao City, including eligible students from Jiangxi Medical College who had lived in Shangrao for at least 6 months. Eligible participants were Shangrao residents who had lived in the city for at least 6 months and were aged 5 years or older. Various recruitment activities were carried out in communities, households, schools, and factories to collect questionnaires from diverse population groups. Additionally, quick response (QR) codes were disseminated via the official WeChat account of “Shangrao Disease Control” and through work-related and school-related WeChat groups, encouraging residents and college students to voluntarily scan and complete the questionnaire. Because recruitment channel was not recorded as an individual-level questionnaire field, exact channel-specific response proportions could not be calculated.

No formal demographic quotas were prespecified by age, sex, place of residence, occupation, or educational attainment. The recruitment strategy aimed to maximize geographic coverage and include participants from different daily-life and occupational settings, rather than to generate a sample proportionally matched to the underlying population. Therefore, the achieved sample should be regarded as a convenience sample with broad geographic coverage, rather than a probability sample fully representative of the resident population of Shangrao. As reported in the Results and noted in the Discussion, women, adults aged 20–49 years, and respondents with junior college or undergraduate education were over-represented, whereas older adults and individuals with lower educational attainment were under-represented.

A total of 2,119 individuals were surveyed, including teachers, students, workers, farmers, medical staff, and retirees. Field investigators provided standardized explanations regarding the study purpose, the definition of contact, and the questionnaire completion procedures. For young children aged <12 years, as well as older adults or visually impaired participants who were unable to complete the questionnaire independently, the questionnaire was completed by their parents, adult family members, or community healthcare workers, who entered the information provided by the participant. Because recruitment was anonymous, individual-level information on those who declined participation was not collected. Informally reported reasons for refusal during recruitment mainly included time constraints and privacy concerns. No monetary or material incentives were offered. Participation was voluntary and informed consent was obtained on the questionnaire’s landing page.

### 2.2 Questionnaire design

The questionnaire survey was conducted in an online format. The opening section of the questionnaire included greetings, a statement of the study purpose, assurances regarding data confidentiality, and an emphasis that participants were required to report all individuals with whom they had contact during the 24-h period from 00:00 to 24:00 of the previous day. Contact was defined as either a two-way conversation involving three or more words (e.g., “Hi!,” “Hello,” “How are you?,” “Well, how about you?”) or physical skin contact (e.g., handshakes, hugs, kisses, or other direct contact activities). No additional duration threshold was applied: both brief encounters and sustained conversations were counted as contacts as long as they met the above definition of a three-word two-way exchange or physical skin contact. Contact duration was recorded categorically in the questionnaire but was not used as a primary stratification variable in the present analysis. Each individual was counted once per day, regardless of how many times the respondent had contact with that person on the previous day. Online-only interactions, such as phone calls, video calls, and instant-message exchanges, were excluded; only in-person three-word two-way exchanges or physical skin contact were counted as contacts. The “previous day” referred to the calendar day immediately preceding questionnaire submission and could therefore be a working day, weekend day, or holiday. The basic information collected from respondents included gender, age, area of residence, radius of activity on the previous day, educational attainment, and the location of their usual life, study, or work. Contacts reported by respondents were classified into household contacts and non-household contacts. Separate information was collected for each category. For household contacts, data were collected on the number of household members, the relationship between each household member and the respondent, contact duration, age, and mode of contact. For non-household contacts, respondents were asked to report all individuals encountered from 00:00 to 24:00 of the previous day other than household members. They were required to provide the number of such contacts, the relationship with the respondent, contact duration, age, and mode of contact. The full questionnaire is provided as Supplementary File S1.

### 2.3 Data collection

The data collection period was from April 2025 to September 2025. A total of 2,119 questionnaires were collected. After excluding invalid questionnaires due to incomplete information, 2,101 valid questionnaires were retained for analysis. All valid responses were submitted in 2025, including 403 in April, 1,513 in May, and 185 in September. Thus, most responses were collected during the spring school term, with a smaller proportion collected at the beginning of the autumn school term. Among these reported contact days, 2,007 were weekdays and 94 were weekend days. Given that the data were collected within a relatively short period in 2025 and that the weekend and autumn-term subsamples were small, we pooled all valid observations for the main analysis to preserve statistical stability. Therefore, the estimates should be interpreted primarily as school-term, weekday-dominant contact patterns rather than as annual average contact patterns.

To minimize repeated submissions, the Wenjuanxing online questionnaire platform was configured to restrict each WeChat account and device to one submission only. The questionnaire landing page also informed participants that each respondent should complete the survey only once. After data collection, the dataset was further checked for potential duplicate records based on available respondent information and submission characteristics, and no duplicated valid questionnaires were identified.

### 2.4 Quality control

The definition of contact and the content of the questionnaire were explicitly presented at the beginning of the survey as follows: “Respondents are requested to report all individuals with whom they had contact during the 24-h period from 00:00 to 24:00 of the previous day.” The questionnaire was designed to be straightforward, using multiple-choice questions to collect basic respondent information. For potentially confusing items, yellow shading was applied to highlight and prompt respondents. Explanatory notes were provided below the questions to clarify completion requirements, for example: “How many people do you live with?” (0 indicates living alone, 1 indicates having a roommate or family member, and so on), “Information of household members:” (write one line per person; click “Continue filling” to add more if needed), “Total number of people you had contact with from 00:00 to 24:00 yesterday:” (excluding household members). These measures were implemented to enhance the quality of questionnaire completion.

### 2.5 Data processing and statistical analysis

Data were organized using Microsoft Office Excel 2019. Unweighted descriptive analyses were performed using SPSS version 27.0, with a two-sided significance level of *α* = 0.05. The arithmetic mean and 95% confidence interval were used to describe the daily number of contacts. Differences between two groups were assessed using the t-test, and differences among multiple groups were assessed using one-way analysis of variance (ANOVA). When ANOVA results were statistically significant, *post hoc* pairwise comparisons were conducted using the Student–Newman–Keuls (SNK) method or Tamhane’s T2 method, depending on the homogeneity of variance test.

A main-effects general linear model was first fitted as a preliminary multivariable analysis for comparability with the descriptive analyses (Table 1). Because daily contact counts are non-negative integer-valued outcomes and showed overdispersion, the principal multivariable analysis was performed using a negative binomial regression model with a log link. The daily total number of contacts was specified as the outcome, and sex, age group, county/district, urban/rural residence, mobility range, and educational attainment were entered simultaneously as fixed effects. Results are reported as incidence rate ratios (IRRs) with 95% Wald confidence intervals (Table 2).

**Table 1:** Main-effects general linear model for daily total contacts.

Dependent variable	Source	Type III sum of squares	df	Mean square	*F*	*p*-value
Total contacts	Corrected model	2543.952	26	97.844	4.430	<0.001
	Gender	146.405	1	146.405	6.628	0.010
	Educational attainment	44.249	4	11.062	0.501	0.735
	District/county	467.780	11	42.525	1.925	0.032
	Mobility range	382.033	3	127.344	5.765	<0.001
	Residence	8.104	1	8.104	0.367	0.545
	Age group	1561.061	6	260.177	11.779	<0.001
	a, *R*^2^ = 0.053 (adjusted *R*^2^ = 0.041)					

**Table 2:** Unweighted negative binomial regression of daily total number of contacts on participant characteristics.

Variable/category	IRR	95% CI	*p*-value
Sex (ref: female)			
Male	1.081	(1.02, 1.15)	0.014
Age group, years (ref: 20–29)			
5–19	1.740	(1.51, 2.00)	<0.001
30–39	1.148	(1.06, 1.24)	<0.001
40–49	1.151	(1.06, 1.25)	<0.001
50–59	1.060	(0.96, 1.18)	0.264
60–69	1.030	(0.80, 1.32)	0.819
≥70	0.783	(0.43, 1.44)	0.429
County/district (ref: Xinzhou)			
Wuyuan	1.046	(0.90, 1.21)	0.546
Guangfeng	1.250	(1.05, 1.48)	0.010
Guangxin	1.097	(0.93, 1.29)	0.265
Yushan	1.212	(1.04, 1.41)	0.014
Yanshan	1.086	(0.92, 1.28)	0.340
Hengfeng	1.037	(0.88, 1.23)	0.670
Yiyang	1.167	(0.98, 1.38)	0.074
Dexing	1.177	(1.02, 1.36)	0.026
Wannian	1.193	(1.02, 1.39)	0.027
Poyang	1.187	(1.01, 1.39)	0.032
Yugan	1.097	(0.92, 1.32)	0.317
Residence (ref: urban)			
Rural	1.024	(0.96, 1.09)	0.454
Mobility range (ref: ≤1 km)			
1–3 km	1.042	(0.95, 1.14)	0.359
4–10 km	1.134	(1.05, 1.23)	0.002
>10 km	1.179	(1.08, 1.29)	<0.001
Educational attainment (ref: junior college/undergrad.)			
Primary school and below	1.117	(0.81, 1.54)	0.501
Junior high school	0.872	(0.69, 1.11)	0.264
Senior high/vocational	0.998	(0.93, 1.07)	0.946
Master’s degree and above	0.922	(0.67, 1.27)	0.622

IRR, incidence rate ratio; CI, confidence interval. Reference categories: 20–29 years (age group); Xinzhou District (county/district); urban (residence); ≤1 km (mobility range); junior college/undergraduate (educational attainment); female (sex). Model fitted in Python (statsmodels) using a log link.

To assess the robustness of the findings to the imbalanced sample composition, post-stratification weighting was conducted using the 2020 Seventh National Population Census of Shangrao City as the reference population. Weights were generated by iterative proportional fitting and normalized to the achieved sample size of 2,101. Two primary weighting specifications, age × sex and county × sex, were retained for sensitivity analyses; a joint age × county × sex specification was attempted but was not used because of sparse or structurally empty cells. Weighted descriptive estimates and weighted negative binomial regressions were then compared with the unweighted results (Table 3). The weighting analyses were performed in Python 3.11 using pandas and statsmodels.

**Table 3:** Side-by-side comparison of incidence rate ratios for the mean daily total number of contacts.

Variable/category	Unweighted IRR (95% CI)	Age × sex (trimmed) IRR (95% CI)	County × sex (trimmed) IRR (95% CI)	*p*-value (unweighted/age × sex weighted/county × sex weighted)
Sex (ref: female)				
Male	1.081 (1.02, 1.15)	1.003 (0.95, 1.06)	1.030 (0.97, 1.09)	0.014/0.922/0.296
Age group, years (ref: 20–29)				
5–19	1.740 (1.51, 2.00)	1.623 (1.41, 1.87)	1.713 (1.48, 1.98)	<0.001/<0.001/<0.001
30–39	1.148 (1.06, 1.24)	1.182 (1.08, 1.29)	1.227 (1.13, 1.33)	<0.001/<0.001/<0.001
40–49	1.151 (1.06, 1.25)	1.208 (1.10, 1.32)	1.289 (1.19, 1.40)	<0.001/<0.001/<0.001
50–59	1.060 (0.96, 1.18)	1.129 (1.00, 1.27)	1.208 (1.09, 1.33)	0.264/0.040/<0.001
60–69	1.030 (0.80, 1.32)	1.038 (0.94, 1.15)	0.826 (0.68, 1.00)	0.819/0.460/0.050
≥70	0.783 (0.43, 1.44)	0.749 (0.62, 0.90)	1.069 (0.42, 2.71)	0.429/0.003/0.887
County/district (ref: Xinzhou)				
Wuyuan	1.046 (0.90, 1.21)	0.908 (0.79, 1.04)	1.036 (0.86, 1.24)	0.546/0.179/0.702
Guangfeng	1.250 (1.05, 1.48)	1.061 (0.89, 1.27)	1.157 (1.00, 1.34)	0.010/0.513/0.055
Guangxin	1.097 (0.93, 1.29)	0.933 (0.79, 1.10)	1.029 (0.88, 1.20)	0.265/0.420/0.723
Yushan	1.212 (1.04, 1.41)	1.073 (0.92, 1.25)	1.192 (1.03, 1.38)	0.014/0.367/0.021
Yanshan	1.086 (0.92, 1.28)	0.885 (0.75, 1.05)	1.043 (0.88, 1.23)	0.340/0.163/0.623
Hengfeng	1.037 (0.88, 1.23)	0.829 (0.72, 0.96)	1.095 (0.90, 1.33)	0.670/0.012/0.361
Yiyang	1.167 (0.98, 1.38)	1.102 (0.94, 1.30)	1.166 (0.97, 1.40)	0.074/0.242/0.094
Dexing	1.177 (1.02, 1.36)	1.091 (0.95, 1.26)	1.139 (0.95, 1.36)	0.026/0.226/0.151
Wannian	1.193 (1.02, 1.39)	0.969 (0.83, 1.13)	1.173 (0.99, 1.39)	0.027/0.691/0.071
Poyang	1.187 (1.01, 1.39)	0.892 (0.77, 1.04)	1.218 (1.06, 1.40)	0.032/0.143/0.006
Yugan	1.097 (0.92, 1.32)	0.945 (0.78, 1.14)	1.010 (0.87, 1.17)	0.317/0.563/0.900
Residence (ref: urban)				
Rural	1.024 (0.96, 1.09)	1.104 (1.04, 1.18)	1.026 (0.96, 1.09)	0.454/0.002/0.421
Mobility range (ref: ≤1 km)				
1–3 km	1.042 (0.95, 1.14)	1.075 (0.99, 1.16)	1.006 (0.92, 1.10)	0.359/0.070/0.899
4–10 km	1.134 (1.05, 1.23)	1.127 (1.04, 1.22)	1.080 (0.99, 1.17)	0.002/0.002/0.068
>10 km	1.179 (1.08, 1.29)	1.186 (1.08, 1.30)	1.179 (1.08, 1.29)	<0.001 / <0.001 / <0.001
Educational attainment (ref: junior college/undergrad.)				
Primary school and below	1.117 (0.81, 1.54)	1.179 (1.04, 1.34)	0.829 (0.58, 1.19)	0.501/0.013/0.309
Junior high school	0.872 (0.69, 1.11)	0.727 (0.60, 0.88)	0.749 (0.58, 0.97)	0.264 / <0.001/0.031
Senior high/vocational	0.998 (0.93, 1.07)	1.048 (0.98, 1.12)	0.996 (0.93, 1.06)	0.946/0.164/0.907
Master’s degree and above	0.922 (0.67, 1.27)	0.936 (0.65, 1.35)	0.958 (0.64, 1.43)	0.622/0.723/0.832

Reference categories: 20–29 years; Xinzhou District; urban; ≤1 km; junior college/undergraduate; female. The age × sex and county × sex trimmed weighting specifications are described in Section 2.5.

**Figure 1:**
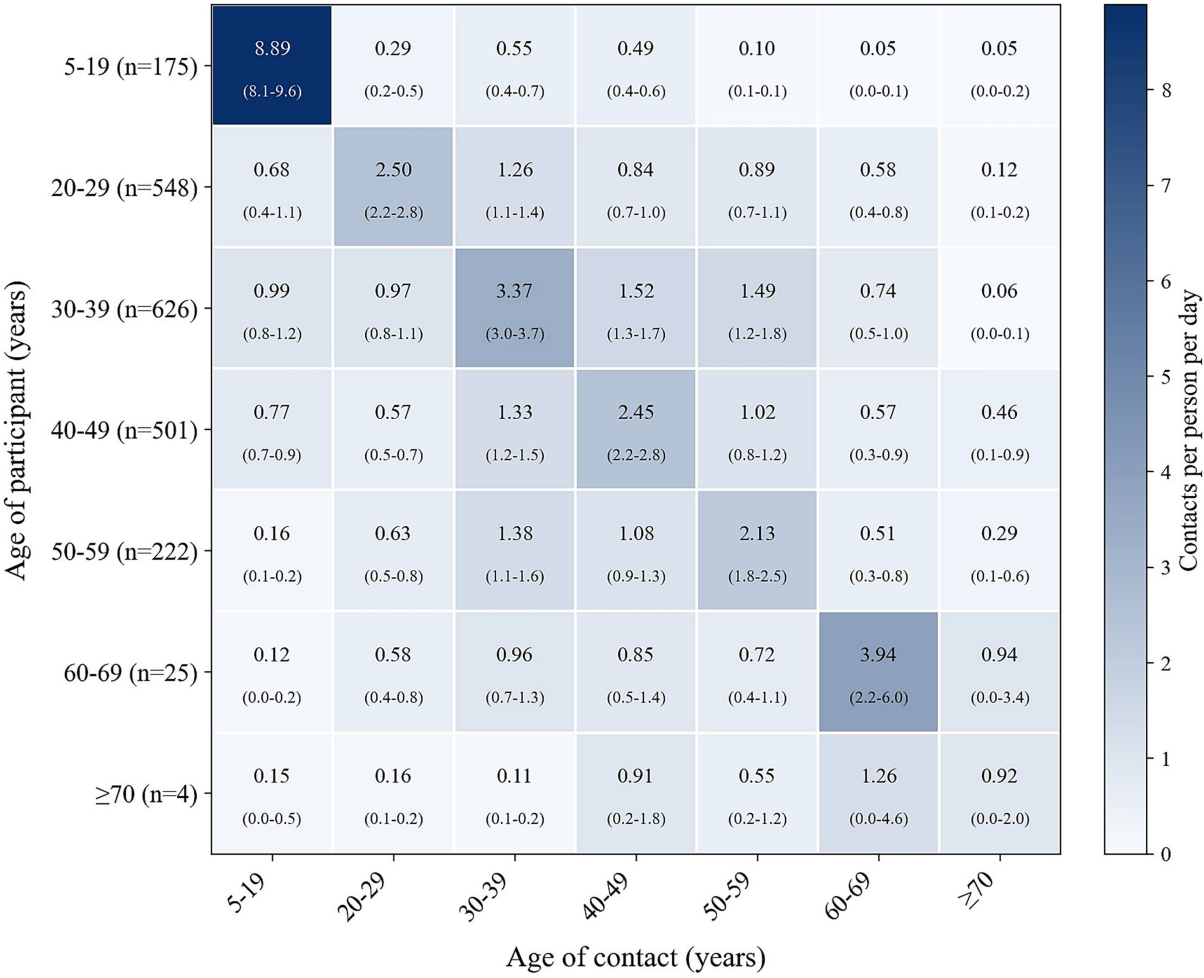
Overall age-by-age contact matrix.

The age-specific per-capita contact matrix was constructed following the approach used by Zhang et al. in the Shanghai social contact study (7), in which the matrix was made reciprocal and population-age weighted using the socialmixr framework. Specifically, we used the 2020 Shangrao census age structure to enforce reciprocity such that C_ij_N_i_ = C_ji_N_j_; 95% confidence intervals were obtained by bootstrap resampling of participants (Figure 1).

Box plots, heat maps, and age-specific contact matrices were generated using R version 4.5.1 and Python 3.11 to visualize contact distributions across demographic groups, county/districts, settings, and respondent-contact age groups.

## 3 Results

### 3.1 Demographic characteristics of the respondents

Among the 2,101 respondents, 556 (26.46%) were male and 1,545 (73.54%) were female. The age distribution was predominantly concentrated in the 20–49 years range. Of the total sample, 57.83% resided in urban areas and 42.17% in rural areas. The county with the largest proportion of respondents was Wuyuan County, accounting for 16.66% of the sample. Regarding mobility range, 26.13% of respondents reported a radius of ≤1 km, 16.80% reported 1–3 km, 40.36% reported 4–10 km, and 16.71% reported >10 km. With respect to educational attainment, 0.62% had completed primary school or below, 1.76% junior high school, 20.94% senior high school or technical secondary school, 75.77% junior college or undergraduate education, and 0.90% had a master’s degree or above (Table 4).

**Table 4:** Distribution of mean daily total, household, and non-household contacts among respondents by demographic characteristics (*n* = 2,101).

Factor	*N* (%)	Total contacts mean (95% CI)	*t*/*F*	*p*-value for total contacts	Household contacts (95% CI)	*p*-value for household contacts	Non-household contacts (95% CI)	*p*-value for non-household contacts
Total	2,101 (100.0)	7.91 (7.71, 8.12)	/	/	2.73 (2.64, 2.81)	/	5.19 (5.01, 5.37)	/
Gender								
Male	556 (26.46)	8.25 (7.82, 8.67)	1.821	0.069	2.58 (2.41, 2.75)	0.040	5.67 (5.30, 6.03)	0.002
Female	1,545 (73.54)	7.80 (7.56, 8.03)			2.78 (2.68, 2.87)		5.02 (4.81, 5.23)	
Age group (years)								
5–19	175 (8.33)	9.99 (9.33, 10.66)	10.252	<0.001	4.88 (4.69, 5.07)	<0.001	5.11 (4.50, 5.72)	0.888
20–29	548 (26.08)	6.95 (6.55, 7.35)			1.87 (1.71, 2.03)		5.08 (4.71, 5.46)	
30–39	626 (29.80)	8.11 (7.73, 8.49)			3.00 (2.85, 3.15)		5.11 (4.78, 5.44)	
40–49	501 (23.85)	8.17 (7.75, 8.59)			2.83 (2.68, 2.98)		5.34 (4.97, 5.71)	
50–59	222 (10.57)	7.66 (7.06, 8.26)			2.23 (2.02, 2.44)		5.43 (4.89, 5.97)	
60–69	25 (1.19)	7.12 (5.42, 8.82)			2.04 (1.53, 2.55)		5.08 (3.56, 6.60)	
≥70	4 (0.19)	5.25 (−0.01, 10.51)			1.25 (0.45, 2.05)		4.00 (−1.03, 9.03)	
Residence								
Urban area	1,215 (57.83)	8.03 (7.76, 8.29)	1.267	0.205	2.94 (2.84, 3.04)	<0.001	5.09 (4.86, 5.32)	0.212
Rural area	886 (42.17)	7.76 (7.43, 8.08)			2.43 (2.30, 2.57)		5.33 (5.04, 5.62)	
District/county								
Wuyuan	350 (16.66)	7.16 (6.67, 7.65)	2.199	0.012	2.24 (2.05, 2.44)	<0.001	4.91 (4.48, 5.35)	0.076
Dexing	312 (14.85)	8.01 (7.50, 8.51)			2.52 (2.32, 2.73)		5.48 (5.00, 5.96)	
Wannian	218 (10.38)	8.14 (7.47, 8.81)			2.72 (2.47, 2.97)		5.42 (4.81, 6.02)	
Xinzhou	217 (10.33)	8.60 (7.97, 9.24)			4.02 (3.75, 4.28)		4.59 (4.06, 5.11)	
Guangxin	175 (8.33)	7.60 (6.83, 8.37)			2.49 (2.21, 2.77)		5.11 (4.44, 5.79)	
Poyang	148 (7.04)	8.41 (7.61, 9.20)			2.51 (2.21, 2.81)		5.90 (5.19, 6.60)	
Yushan	147 (7.00)	8.54 (7.80, 9.29)			2.80 (2.50, 3.09)		5.75 (5.09, 6.41)	
Yiyang	128 (6.09)	7.99 (7.10, 8.88)			2.90 (2.55, 3.25)		5.09 (4.32, 5.87)	
Guangfeng	110 (5.24)	8.49 (7.52, 9.46)			3.14 (2.73, 3.54)		5.35 (4.54, 6.17)	
Yanshan	105 (5.00)	7.55 (6.71, 8.39)			2.68 (2.31, 3.04)		4.88 (4.10, 5.65)	
Hengfeng	104 (4.95)	7.11 (6.22, 7.99)			2.37 (2.06, 2.67)		4.74 (3.97, 5.51)	
Yugan	87 (4.14)	7.66 (6.66, 8.65)			2.63 (2.24, 3.02)		5.02 (4.17, 5.88)	
Mobility range								
≤1 km	549 (26.13)	7.64 (7.23, 8.05)	3.398	0.017	2.82 (2.64, 3.01)	0.381	4.82 (4.46, 5.18)	0.003
1–3 km	353 (16.80)	7.48 (7.00, 7.96)			2.67 (2.46, 2.87)		4.81 (4.38, 5.24)	
4–10 km	848 (40.36)	8.03 (7.71, 8.36)			2.66 (2.54, 2.78)		5.38 (5.09, 5.66)	
>10 km	351 (16.71)	8.49 (7.99, 8.99)			2.79 (2.61, 2.97)		5.70 (5.25, 6.14)	
Educational attainment								
Primary school and below	13 (0.62)	7.23 (5.43, 9.03)	0.866	0.484	1.46 (0.99, 1.93)	0.031	5.77 (3.97, 7.57)	0.416
Junior high school	37 (1.76)	6.68 (5.03, 8.32)			2.43 (1.85, 3.02)		4.24 (2.94, 5.55)	
Senior high/vocational school	440 (20.94)	7.85 (7.41, 8.30)			2.88 (2.71, 3.06)		4.97 (4.57, 5.37)	
Junior college/undergraduate	1,592 (75.77)	7.97 (7.74, 8.21)			2.70 (2.61, 2.80)		5.27 (5.06, 5.48)	
Master’s degree and above	19 (0.90)	7.21 (4.51, 9.91)			2.26 (1.26, 3.26)		4.95 (2.63, 7.27)	

### 3.2 Distribution of daily average number of contacts across different population groups

A total of 2,101 respondents reported contact with 16,629 individuals. Among these, 5,726 were household contacts and 10,903 were non-household contacts. The mean daily number of contacts was 7.91, comprising 2.73 household contacts and 5.19 non-household contacts (Table 4).

#### 3.2.1 Gender

The mean daily number of contacts was 8.25 for males and 7.80 for females. An independent sample *t*-test showed no statistically significant difference in total contacts between genders (*t* = 1.821, *p* = 0.069). With respect to household contacts, the mean daily number was 2.58 for males and 2.78 for females; this difference was statistically significant (*p* = 0.040). For non-household contacts, the mean daily number was 5.67 for males and 5.02 for females; this difference was also statistically significant (*p* = 0.002) (Table 4).

#### 3.2.2 Age group

Analysis by age group revealed that the mean daily number of contacts was highest among the 5–19 years age group (9.99), followed by the 40–49 years age group (8.17) (Table 4). One-way ANOVA indicated statistically significant differences in the mean daily number of total contacts across age groups (*p* < 0.01). With respect to household contacts, the 5–19 years age group also had the highest mean daily number (4.88), and the differences across age groups were statistically significant (*p* < 0.01). For non-household contacts, the 50–59 years age group had the highest mean daily number (5.43); however, the differences across age groups were not statistically significant (*p* = 0.888).

*Post hoc* pairwise comparisons using the SNK method were conducted for total contacts and household contacts across age groups. For total contacts, the ≥70 years age group had a significantly lower contact frequency than the 5–19 years age group, whereas no statistically significant differences were observed among the other age groups. For household contacts, the 5–19 years adolescent group had a significantly higher contact frequency than all other age groups, forming an independent subset. The adult population aged 20–59 years exhibited intermediate contact frequencies. A pattern of continuity was observed: the contact frequencies of the 20–29, 60–69, and 50–59 years age groups did not differ significantly from the lowest-contact age group (≥70 years), nor did they differ significantly from the higher 30–39 and 40–49 years age groups. Because the numbers of respondents aged 60–69 years (*N* = 25) and ≥70 years (*N* = 4) were small, estimates and *post hoc* comparisons involving these older age groups should be interpreted cautiously and regarded as descriptive rather than definitive evidence of age-specific differences (Tables 4, 5).

**Table 5:** Post-hoc multiple comparisons of mean daily contacts across age groups (SNK method).

Mean daily number of contacts					
Contact category	Age group	*N*	Subset for *α* = 0.05		
			Homogeneous subset A	Homogeneous subset B	Homogeneous subset C
Total contacts	5–19	175		9.99	
	20–29	548	6.95	6.95	
	30–39	626	8.11	8.11	
	40–49	501	8.17	8.17	
	50–59	222	7.66	7.66	
	60–69	25	7.12	7.12	
	≥70	4	5.25		
	*p*-value		0.295	0.251	
Household contacts	5–19	175			4.88
	20–29	548	1.87	1.87	
	30–39	626		3.00	
	40–49	501		2.83	
	50–59	222	2.23	2.23	
	60–69	25	2.04	2.04	
	≥70	4	1.25		
	*p*-value			0.251	1.000

Values in the same column do not differ significantly at *α* = 0.05 by the Student–Newman–Keuls test. *p*-value (within-subset homogeneity) is the probability that, within a homogeneous subset, the group means do not differ.

#### 3.2.3 Place of residence

Analysis by residential area showed that the mean daily number of total contacts was 8.03 in urban areas and 7.76 in rural areas. An independent sample *t*-test indicated no statistically significant difference between the two groups (*t* = 1.267, *p* = 0.205). For household contacts, the mean daily number was 2.94 in urban areas and 2.43 in rural areas, and this difference was statistically significant (*p* < 0.01). For non-household contacts, the mean daily number was 5.09 in urban areas and 5.33 in rural areas; this difference was not statistically significant (*p* = 0.212) (Table 4).

Descriptive analysis by county/district showed that the mean daily number of total contacts was highest in Xinzhou District (8.60), followed by Yushan County (8.54), Guangfeng District (8.49), and Poyang County (8.41). One-way ANOVA indicated an overall difference in total contacts across counties/districts (*F* = 2.199, *p* = 0.012). For household contacts, Xinzhou District also had the highest mean daily number (4.02), and the overall difference across counties/districts was statistically significant (*p* < 0.01). For non-household contacts, Poyang County had the highest mean daily number (5.90), but the overall difference across counties/districts did not reach statistical significance (*p* = 0.076). Because the assumption of homogeneity of variance was violated for total contacts across counties/districts, Tamhane’s T2 method was used for *post hoc* multiple comparisons. After post hoc comparison, only the difference in total contacts between Xinzhou District and Wuyuan County remained statistically significant, whereas the other pairwise comparisons were not significant (Table 6). Therefore, the observed county/district heterogeneity should be interpreted cautiously and should not be regarded as evidence of broad or stable differences across all counties/districts.

**Table 6:** Post-hoc multiple comparisons of total daily contacts by district (Tamhane’s T2 method) (abridged table).

Contact category	(I) District	(J) District	Mean diff (I-J)	STD. error	Sig.	95% CI	
						95% CI lower	95% CI upper
Total contacts	Wuyuan	Dexing	−0.849	0.360	0.708	−2.06	0.36
		Wannian	−0.980	0.422	0.749	−2.41	0.45
		Xinzhou	−1.447*	0.407	0.028	−2.82	−0.07
		Guangxin	−0.443	0.465	1.000	−2.02	1.13
		Poyang	−1.248	0.473	0.444	−2.86	0.36
		Yushan	−1.387	0.451	0.143	−2.92	0.15
		Yiyang	−0.835	0.515	0.999	−2.59	0.92
		Guangfeng	−1.334	0.549	0.658	−3.21	0.54
		Yanshan	−0.395	0.492	1.000	−2.08	1.29
		Hengfeng	0.051	0.513	1.000	−1.70	1.81
		Yugan	−0.498	0.558	1.000	−2.42	1.42
	Xinzhou	Wuyuan	1.447*	0.407	0.028	0.07	2.82
		Dexing	0.597	0.412	1.000	−0.80	1.99
		Wannian	0.466	0.468	1.000	−1.12	2.05
		Guangxin	1.004	0.507	0.962	−0.71	2.72
		Poyang	0.198	0.515	1.000	−1.55	1.95
		Yushan	0.059	0.495	1.000	−1.62	1.74
		Yiyang	0.611	0.554	1.000	−1.27	2.50
		Guangfeng	0.113	0.585	1.000	−1.88	2.11
		Yanshan	1.051	0.532	0.964	−0.76	2.86
		Hengfeng	1.498	0.551	0.377	−0.38	3.38
		Yugan	0.949	0.593	1.000	−1.08	2.98
* The significance level of the mean difference is 0.05.							

Full pairwise comparison matrix is given in Supplementary Table S1.

#### 3.2.4 Mobility ranges

Analysis by mobility range revealed that the mean daily number of total contacts was highest among respondents with a mobility range exceeding 10 km (8.49), followed by those with ranges of 4–10 km (8.03), ≤1 km (7.64), and 1–3 km (7.48). One-way ANOVA indicated statistically significant differences in total contacts across mobility range groups (*F* = 3.398, *p* = 0.017). *Post hoc* pairwise comparisons using the SNK method showed that when the >10 km group was excluded, the differences among the remaining groups were not statistically significant. Furthermore, no statistically significant difference was observed in total contacts between the 4–10 km and >10 km groups (Tables 4, 7).

**Table 7:** Post-hoc multiple comparisons of mean daily contacts by mobility range (SNK method).

Contact category	Mobility range	*N*	Subset for *α* = 0.05	
			1	2
Total contacts	1–3 km	353	7.48	
	≤1 km	549	7.64	
	4–10 km	848	8.03	8.03
	>10 km	351		8.49
	P value		0.187	0.151
Non-household contacts	1–3 km	353	4.81	
	≤1 km	549	4.82	
	4–10 km	848	5.38	5.38
	>10 km	351		5.70
	P value		0.108	0.247

Values in the same column do not differ significantly at *α* = 0.05 by the Student–Newman–Keuls test. *p*-value (within-subset homogeneity) is the probability that, within a homogeneous subset, the group means do not differ.

For household contacts, the mean daily number was highest among respondents with a mobility range of ≤1 km (2.82), followed by those with >10 km (2.79), 1–3 km (2.67), and 4–10 km (2.66). One-way ANOVA showed no statistically significant differences in household contacts across mobility range groups (*p* = 0.381) (Table 4).

For non-household contacts, the mean daily number was highest among respondents with a mobility range exceeding 10 km (5.70), followed by those with ranges of 4–10 km (5.38), ≤1 km (4.82), and 1–3 km (4.81). One-way ANOVA indicated statistically significant differences across mobility range groups (*p* = 0.003). *Post hoc* comparisons using the SNK method revealed that when the >10 km group was excluded, the differences among the remaining groups were not statistically significant. Additionally, no statistically significant difference was observed in non-household contacts between the 4–10 km and >10 km groups (Tables 4, 7).

#### 3.2.5 Educational attainment

Analysis by educational attainment showed that the mean daily number of total contacts was highest among respondents with a junior college or undergraduate degree, followed by those with senior high school or technical secondary school education, primary school or below, master’s degree or above, and junior high school education. One-way ANOVA indicated no statistically significant differences in total contacts across educational attainment groups (*F* = 0.866, *p* = 0.484). For household contacts, the mean daily number was highest among those with senior high school or technical secondary school education, followed by those with junior college or undergraduate degree, junior high school, primary school or below, and master’s degree or above. The differences across educational attainment groups were statistically significant (*p* = 0.031). For non-household contacts, the mean daily number was highest among those with primary school or below, followed by those with junior college or undergraduate degree, senior high school or technical secondary school, master’s degree or above, and junior high school. The differences across groups were not statistically significant (*p* = 0.416) (Table 4).

#### 3.2.6 Multivariable analysis

To identify factors associated with the daily total number of contacts, a main-effects general linear model with multiple fixed factors was first fitted as a preliminary analysis for comparability with the descriptive analyses (Table 1). The model included sex, age group, county/district, urban/rural residence, mobility range, and educational attainment as fixed effects, without interaction terms. The corrected model used 26 degrees of freedom and was statistically significant overall (*F* = 4.430, *p* < 0.001), but its explanatory power was limited (*R*^2^ = 0.053; adjusted *R*^2^ = 0.041). In this preliminary linear model, sex (*F* = 6.628, *p* = 0.010), age group (*F* = 11.779, *p* < 0.001), county/district (*F* = 1.925, *p* = 0.032), and mobility range (*F* = 5.765, *p* < 0.001) were statistically associated with daily total contacts, whereas urban/rural residence (*F* = 0.367, *p* = 0.545) and educational attainment (*F* = 0.501, *p* = 0.735) were not (Table 1).

Because daily contact counts are non-negative integer-valued outcomes and showed overdispersion in the sample, the principal multivariable analysis was performed using a negative binomial regression model with a log link. Sex, age group, county/district, urban/rural residence, mobility range, and educational attainment were entered simultaneously as fixed effects, and the results are reported as incidence rate ratios (IRRs) with 95% Wald confidence intervals (Table 2).

In the negative binomial model, age group showed the largest and most consistent association with daily total contacts. Compared with the 20–29 years reference group, participants aged 5–19 years had a higher expected daily number of contacts (IRR 1.74, 95% CI 1.51–2.00, *p* < 0.001); the 30–39 years and 40–49 years groups also had higher expected contact numbers (IRR 1.15 for both groups). A wider mobility range was independently associated with more contacts (4–10 km vs. ≤1 km: IRR 1.13, 95% CI 1.05–1.23, *p* = 0.002; >10 km vs. ≤1 km: IRR 1.18, 95% CI 1.08–1.29, *p* < 0.001). Sex reached nominal statistical significance (IRR 1.08, 95% CI 1.02–1.15, *p* = 0.014), whereas urban/rural residence and educational attainment were not statistically significant. Several county/district contrasts were statistically significant at the nominal 0.05 level, but these comparisons were modest in magnitude and should be interpreted cautiously because multiple county-level contrasts were examined.

Across both pre-specified weighting schemes, the principal age and mobility findings were preserved. Under age × sex trimmed weighting, participants aged 5–19 years still had substantially more contacts than those aged 20–29 years (IRR 1.62, 95% CI 1.41–1.87, *p* < 0.001), and the association with wider mobility range remained significant (>10 km vs. ≤1 km: IRR 1.19, 95% CI 1.08–1.30, *p* < 0.001). Similar results were observed under county × sex trimmed weighting, with elevated contact rates in the 5–19 years group (IRR 1.71, 95% CI 1.48–1.98, *p* < 0.001) and among participants with a mobility range >10 km (IRR 1.18, 95% CI 1.08–1.29, *p* < 0.001).

In contrast, the nominal sex effect observed in the unweighted analysis was attenuated after weighting. The higher contact rate among males in the unweighted model (IRR 1.08, *p* = 0.014) was no longer statistically significant under either age × sex weighting (IRR 1.00, *p* = 0.922) or county × sex weighting (IRR 1.03, *p* = 0.296). County-level contrasts were also less stable: most counties were not significantly different from Xinzhou District under age × sex weighting, whereas Poyang and Yushan remained elevated under county × sex weighting. Educational-attainment estimates became significant only in some weighted models and involved very small subgroups, such as respondents with primary school education or below (*n* = 13); these findings should therefore be interpreted cautiously. A side-by-side comparison of the unweighted and weighted negative binomial regression models is presented in Table 3.

### 3.3 Box plots

**Figure 2:**
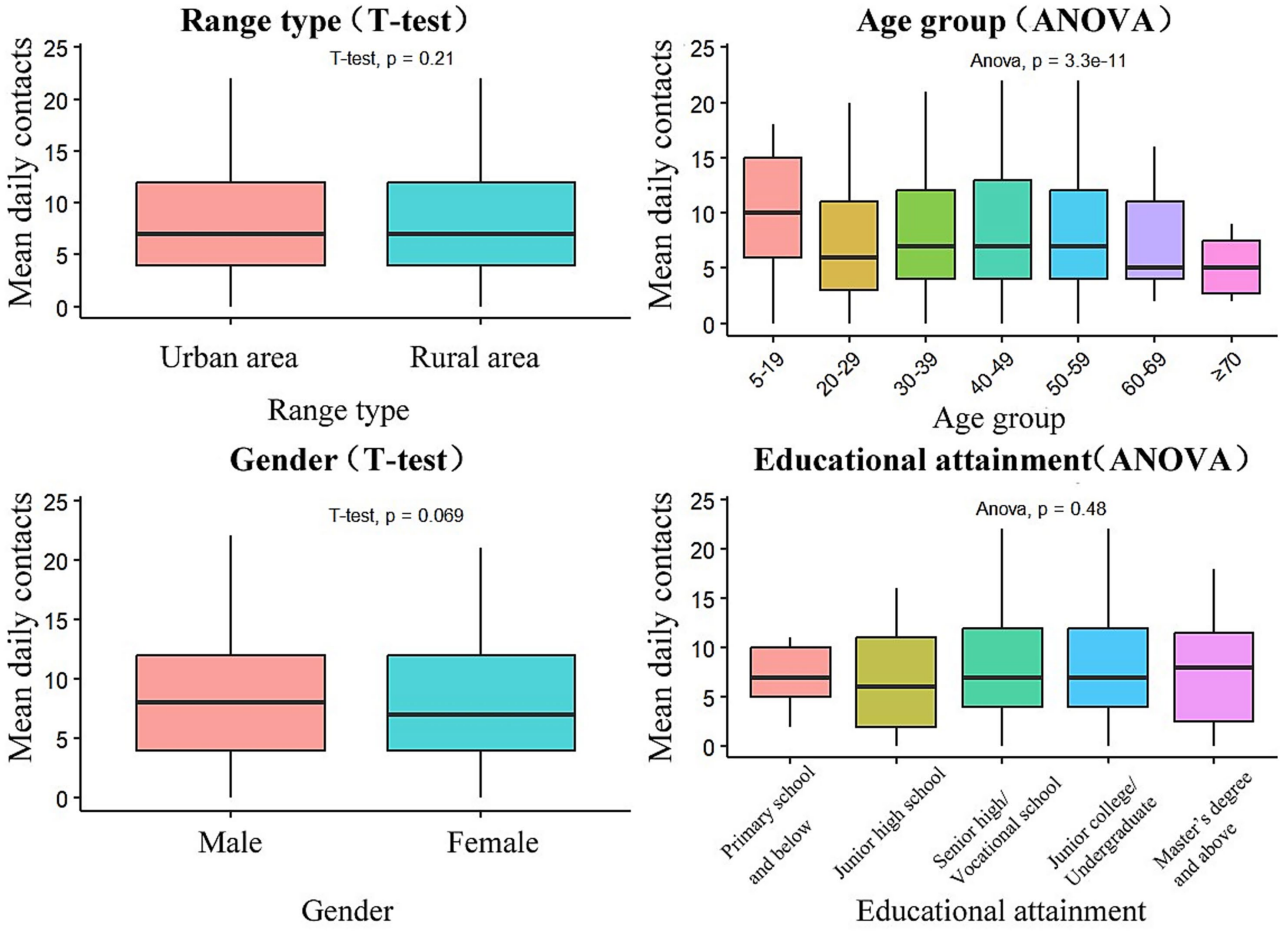
Box plot of mean daily contacts by region, age group, gender and educational attainment.

**Figure 3:**
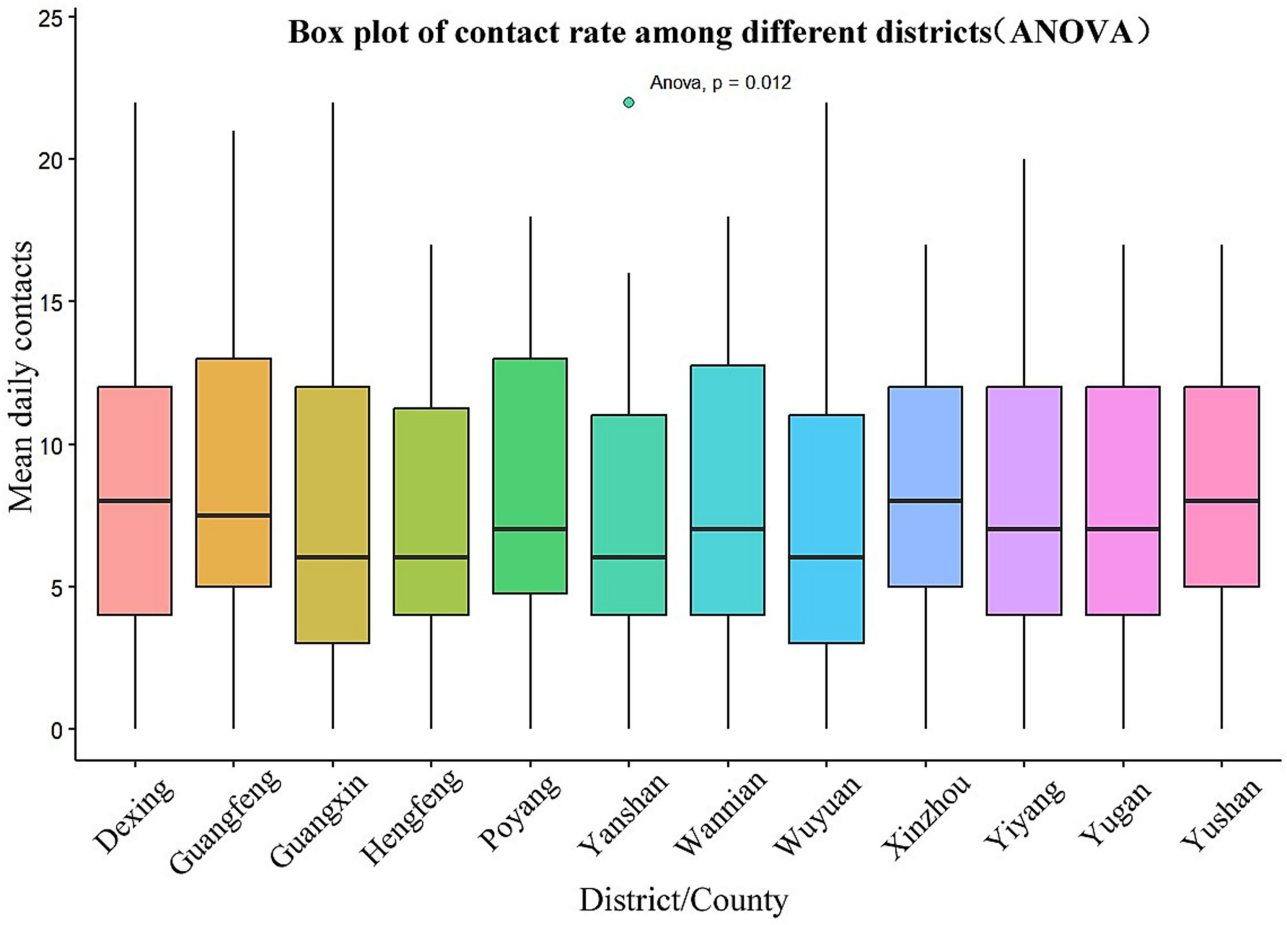
Box plot of mean daily number of contacts among different districts.

Box plots were generated to evaluate the distributional differences in contact frequency across five demographic characteristics: residential area (urban/rural), age group, gender, educational attainment, and county/district of residence. In the binary comparisons of residential area and gender, the results indicated that neither residential area (*p* = 0.21) nor gender (*p* = 0.069) had a statistically significant effect on contact frequency at the *α* = 0.05 level, suggesting no significant difference in the distribution of contact frequencies between urban and rural residents or between males and females. For educational attainment, the analysis of multiple groups yielded a *p* value of 0.48, indicating no statistically significant difference in contact frequency distribution across educational categories. In contrast, the box plots for age group showed a statistically significant difference (*p* < 0.05), with the median contact frequency being relatively higher in the 5–19 years age group and relatively lower in the ≥70 years age group. The box plots for contact frequency across counties/districts also revealed statistically significant differences (*p* = 0.012), indicating heterogeneity in contact frequency distribution by geographic location (Figures 2, 3).

### 3.4 Heat maps

#### 3.4.1 Mean contacts by district/county and age group

**Figure 4:**
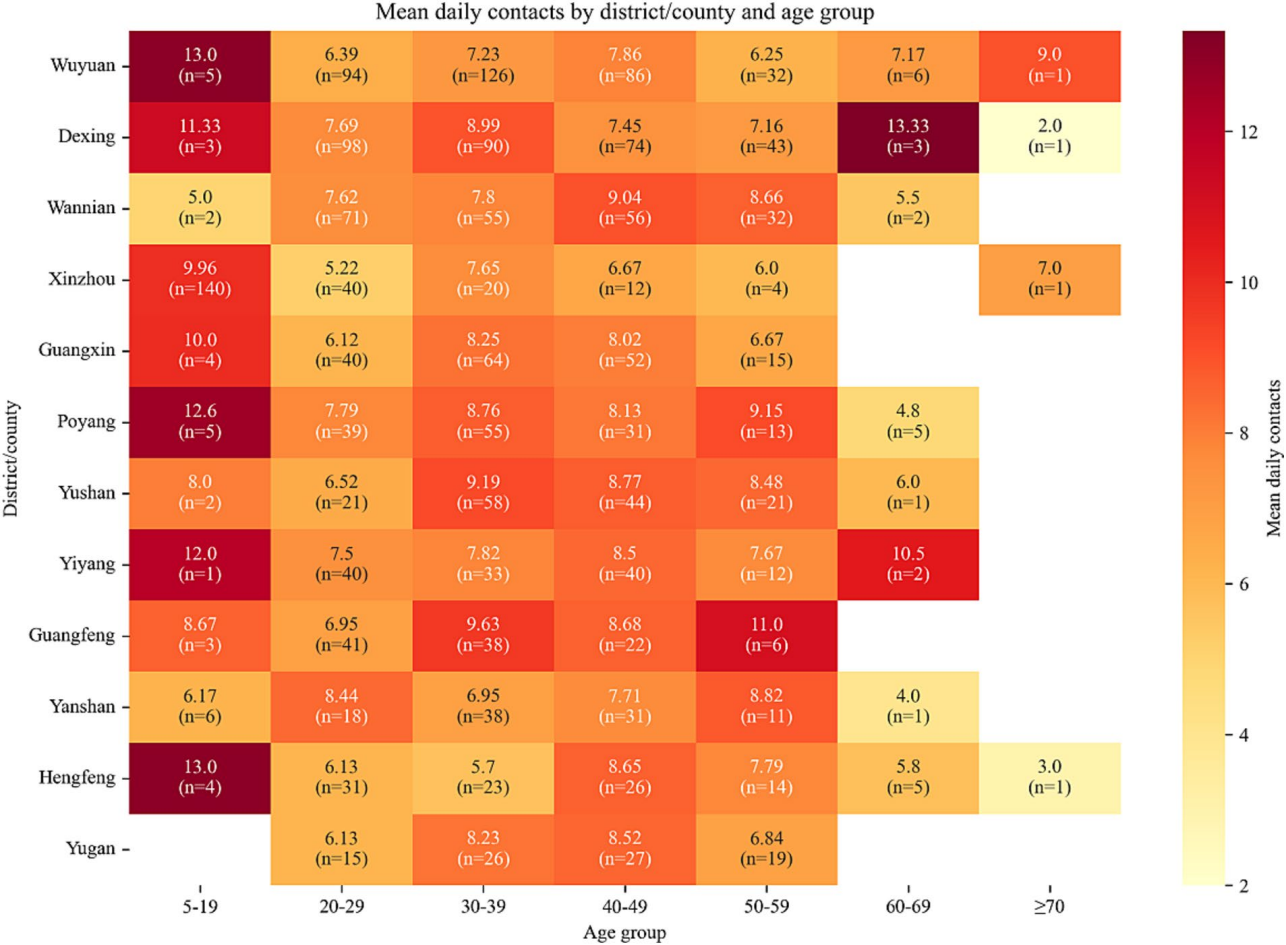
Heat map of mean daily contacts by district/county and age group. Blank cells indicate no respondents/no available observations in that subgroup.

A heat map was constructed to illustrate the distribution of mean daily contact frequency across 12 districts/counties and 7 age groups. Color intensity and numerical values correspond to the magnitude of contact frequency, while the values in parentheses indicate subgroup sample sizes. Overall, the mean daily contact frequency varied across both districts/counties and age groups. The 5–19 years age group showed relatively high contact frequency in several districts/counties, particularly in Wuyuan, Poyang, Yiyang, and Hengfeng; however, this pattern was not consistent across all areas. Some adult age groups also exhibited high contact levels, such as the 50–59 years group in Guangfeng and the 40–49 years group in Wannian. The 60–69 years age group showed an exceptionally high mean contact frequency in Dexing, which should be interpreted cautiously because of the very small sample size in this subgroup (*n* = 3). In contrast, relatively low mean contact frequencies were observed in some older age groups, especially among participants aged ≥70 years in Dexing and Hengfeng, although these estimates were also based on very limited sample sizes. The 20–59 years age groups generally showed moderate and relatively stable contact frequencies across most districts/counties, with values commonly ranging from approximately 6.0 to 9.0. Given the sparse data and small sample sizes in several older age subgroups, the contact patterns among older adults should be interpreted with caution and warrant further investigation (Figure 4).

#### 3.4.2 Mean contacts by study/work/life venue and age group

A heat map was generated to show mean daily contacts across respondents’ usual study, work, or daily-life venues by age group, with cell-specific sample sizes shown in parentheses. Venue classification was based on the respondent’s self-reported usual place of study, work, or daily life in the questionnaire, rather than on all venues visited during the reported day. Therefore, each respondent contributed to only one venue category in this analysis.

School-setting respondents aged 5–19 years had a high mean number of daily contacts (10.06), consistent with intensive peer interactions among adolescents. Higher values were also observed among school-setting respondents aged 40–49 years and 60–69 years, but these cells had very small sample sizes (*n* = 3 and *n* = 1, respectively) and should be interpreted cautiously. Medical-facility respondents showed relatively stable contact levels across age groups, whereas factory respondents generally had lower and more variable contact levels. Regarding the self-reported usual study/work/life venue, 1,551 respondents (73.8%) reported medical institutions as their usual venue. This category therefore accounted for the majority of observations in the venue-based analysis. The remaining respondents were distributed across schools, factories, communities, households, and other daily-life or work settings. Because the medical-institution category was much larger than the other venue categories, venue-specific results should be interpreted as descriptive rather than representative of the underlying distribution of daily-life settings in Shangrao.

**Figure 5:**
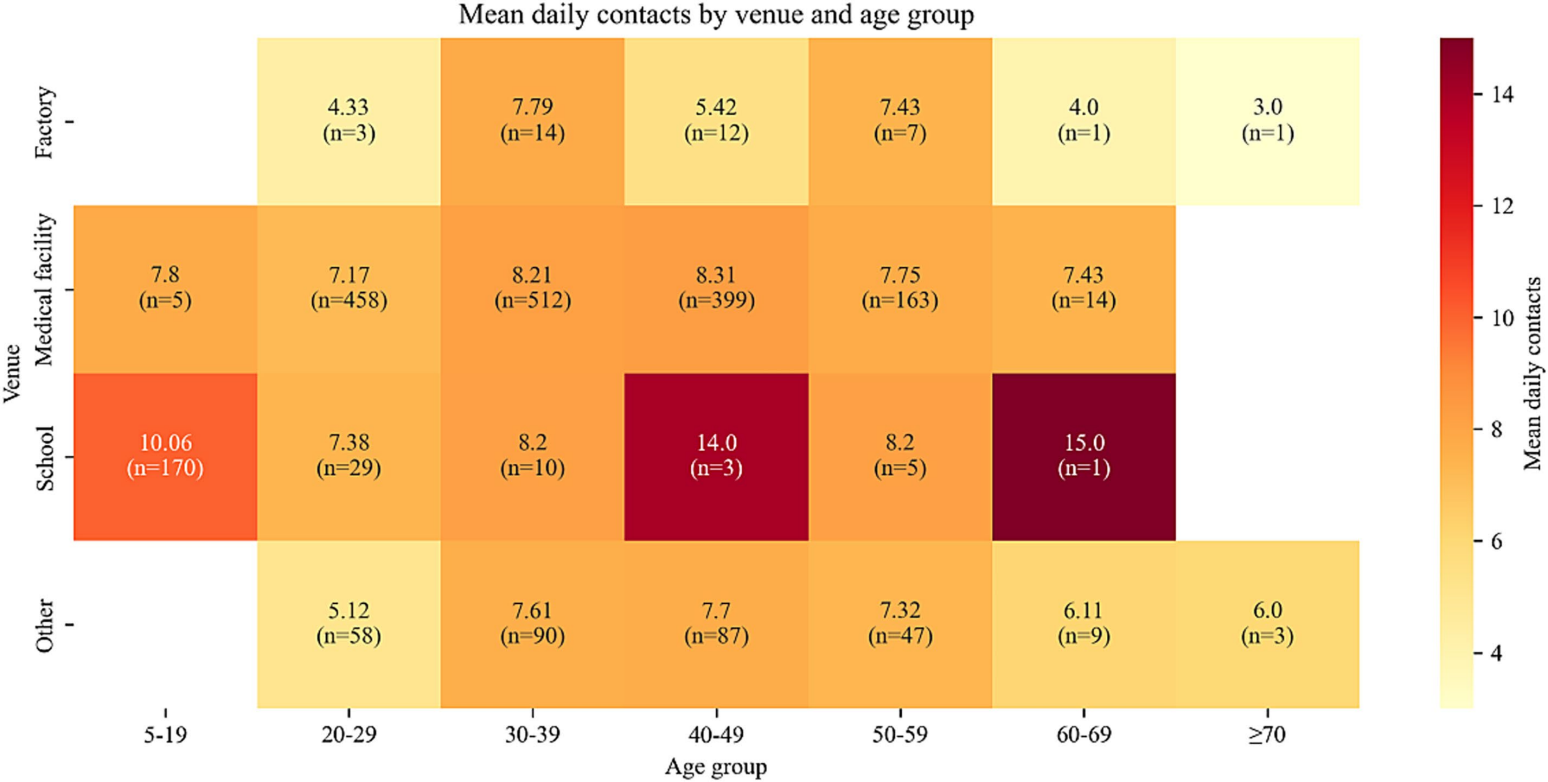
Heat map of mean daily contacts by usual study/work/life venue and age group. Blank cells indicate no respondents/no available observations in that subgroup.

Overall, the venue-by-age heat map suggests that school-related environments are associated with higher contact intensity, particularly among adolescents. However, sparse cells, especially in older age groups, should be regarded as descriptive rather than definitive evidence of venue-specific differences (Figure 5).

### 3.5 Age-by-age contact matrices

The overall age-by-age contact matrix for Shangrao residents is shown in Figure 1. Respondents were stratified into seven age groups (5–19, 20–29, 30–39, 40–49, 50–59, 60–69, ≥70 years). Each cell represents the mean daily number of contacts between participants in the row age group and individuals in the column age group, after the matrix was made population-age weighted and reciprocal (symmetric) using the 2020 Shangrao census age structure (Section 2.5). The colour gradient indicates increasing contact intensity; annotated values show the mean number of contacts with a 95% bootstrap confidence interval, and the number of participants contributing to each row is shown in the row label. Because only four participants were aged ≥70 years, that row rests on few respondents and, although stabilised by the reciprocity correction, should be interpreted with caution.

The matrix showed a clear age-assortative pattern, with higher contact intensity generally observed along the diagonal than in off-diagonal cells. The highest diagonal value was observed among participants aged 5–19 years, indicating frequent contacts with individuals of the same age group, which is consistent with dense peer interactions in school settings. In the adult age groups, contacts between participants aged 30–39 and 40–49 years were relatively prominent, which may reflect workplace and family-based interactions during middle adulthood.

Overall, the matrix suggests that social contact patterns in Shangrao are strongly structured by age, with particularly high within-age contacts among adolescents. These findings support the relevance of age-specific contact information for local transmission modelling and suggest that school-related contacts should be considered when designing targeted interventions for respiratory infectious diseases. Because the numbers of respondents aged 60–69 years and ≥70 years were small, estimates involving older age groups should be interpreted as descriptive and exploratory. The corresponding household and non-household age-by-age contact matrices, constructed with the same weighting and symmetrization, are provided as Supplementary Figures S1, S2.

## 4 Discussion

This study found that the mean daily number of contacts among residents of Shangrao City was 7.91. This estimate is lower than that reported in a survey conducted in Suzhou in 2023(11.51) (4), and also lower than pre-pandemic estimates from Wuhan (14.6) and Shanghai (18.8) (6). These comparisons provide useful context for interpreting contact intensity in Shangrao, but they should not be regarded as direct city-level comparisons because the studies differed in survey timing, pandemic context, sampling strategy, and contact definition. The Suzhou survey was conducted in 2023, shortly after COVID-19 restrictions were lifted, using paper-based stratified sampling, whereas the Wuhan and Shanghai surveys were conducted before the pandemic using face-to-face interviews. In contrast, the present Shangrao survey was conducted in 2025 using a convenience sample recruited through both on-site and online channels. Therefore, the lower mean contact level observed in Shangrao may reflect both contextual differences and methodological heterogeneity, including recruitment mode and sample composition.

With respect to gender differences, this study found no statistically significant difference in total contacts between males and females. However, a significant gender-based divergence emerged in the composition of contact types: males reported significantly more non-household contacts than females, whereas females reported significantly more household contacts than males. This pattern may reflect differences in social and occupational roles, wherein men tend to engage in more workplace and outdoor social activities, while women assume greater responsibility for household care and family interaction. These findings are consistent with the broader literature on gender-specific contact patterns both domestically and internationally (2).

Age emerged as a key factor influencing contact frequency. In this study, the 5–19 years age group had the highest mean daily number of total contacts, and their household contacts were also significantly higher than those of other age groups. This finding aligns closely with results from a study conducted in Suzhou, which similarly reported that the 10–19 years age group had significantly higher contact numbers than other age groups (4). The elevated contact frequency among adolescents is primarily driven by dense social interactions within school settings and high rates of co-residence with family members.

From the perspective of residential area, no significant differences were observed between urban and rural residents in total contacts or non-household contacts, although urban residents reported more household contacts. In the one-way ANOVA and *post hoc* comparison by county/district, Xinzhou District had a higher mean number of total contacts than Wuyuan County. However, other pairwise county/district comparisons were not statistically significant, and county-level contrasts were not stable after multivariable adjustment and post-stratification weighting (Table 3). Therefore, geographic heterogeneity should be interpreted cautiously and regarded as suggestive rather than definitive.

Mobility range had a significant impact on non-household contacts. Respondents with a mobility range exceeding 10 km had the highest mean daily number of total contacts and non-household contacts. This finding suggests that a wider geographic activity radius increases opportunities for interaction with diverse social groups, thereby elevating the risk of cross-regional pathogen transmission. These results reinforce the public health rationale for implementing mobility restrictions during infectious disease outbreaks to mitigate viral spread.

Educational attainment did not significantly influence the mean daily number of contacts among residents in this study. This may imply that social contact patterns in Shangrao City are more strongly determined by biological and social-environmental factors—such as age, occupation, and geographic location—rather than by educational background. This insight supports the development of more precisely targeted public health policies, suggesting that epidemic prevention messaging and interventions should be tailored according to geographic location and age rather than focusing exclusively on specific educational or cultural subgroups.

The negative binomial regression in Table 2 further supported age group as the factor with the most consistent and largest association with daily contact frequency among the measured variables. The 5–19 years group showed the largest increase in expected contact numbers relative to the 20–29 years reference group, and participants with a mobility range greater than 10 km also reported more contacts. These two findings remained consistent in the post-stratification weighted analyses shown in Table 3, suggesting that adolescent congregation and broader daily activity radius are important features to consider in local respiratory infectious disease modelling and targeted prevention planning.

Through heat map visualization, this study further elucidated the structural characteristics of social contacts among Shangrao residents. The heat maps demonstrate that the 5–19 years age group exhibits consistently high contact frequencies across most districts and in school settings, confirming that the school environment constitutes a core transmission setting for respiratory infectious diseases. Notably, elevated mean contact frequencies were also observed among the 40–49 years and 60–69 years age groups in school settings, which may reflect occupational contact patterns associated with parental pick-up/drop-off activities and school staff interactions. In contrast, contact frequencies in factory settings were generally lower and more evenly distributed, suggesting that social density in industrial production environments may be lower than that in educational and social settings. Geographically, the 5–19 years age group represented a high-contact area across all districts, whereas participants aged ≥70 years exhibited markedly low contact levels in districts such as Dexing and Hengfeng. However, this study is limited by the relatively small sample size of participants aged ≥70 years, resulting in considerable variability in some subgroup estimates; therefore, findings pertaining to participants aged ≥70 years should be interpreted with caution. Additionally, contact frequencies in medical settings remained consistently elevated across all age groups, ranging from 7.17 to 8.31, indicating that healthcare facilities represent potential high-risk environments for respiratory pathogen exposure regardless of age.

This study should be interpreted in light of several limitations. Participants were recruited through a combination of on-site invitation and voluntary online completion rather than probability sampling, so the achieved sample was a convenience sample that was not fully random and represents only part of the Shangrao resident population. Certain settings, particularly medical facilities where staff could be enrolled more readily, may be over-represented relative to the general population, and women, adults aged 20–49 years, and respondents with junior-college or undergraduate education were over-represented, whereas young children, older adults, and people with lower educational attainment were under-represented. The contact estimates may therefore be affected by selection toward individuals who were available, willing, and able to complete an online questionnaire, such as those with greater digital literacy. To improve representativeness, future work will strengthen community-level sampling across residential neighbourhoods and broaden recruitment through the official “Shangrao Disease Control” WeChat public account.

Post-stratification weighting against the 2020 Seventh National Population Census was used to assess the robustness of the findings, but this approach has its own limits. The under-representation of young children and older adults produced sparse cells in some strata and prevented the joint age × county × sex specification from converging, and even with trimmed weights the estimates for sparsely represented strata, especially the youngest and oldest age groups, remain exploratory. Because post-stratification weighting can correct only observed marginal imbalances and cannot adjust for unmeasured selection factors such as digital literacy, availability, or willingness to complete an online survey, the weighted results are best regarded as sensitivity analyses rather than fully population-representative estimates.

The timing of data collection was also uneven. Because the survey was administered mainly by CDC staff and field investigators during their working hours, when residents could be reached at schools, workplaces, and communities, the great majority of reported contact days were weekdays (2,007 of 2,101) and only 94 were weekend days. Weekend coverage was inherently limited, as investigators were generally not deployed at weekends or on public holidays, and a respondent approached on a working day reported the preceding day, which was itself usually a weekday. The data were further collected over a short, single-year window from April to September 2025, concentrated in the spring and early-autumn school terms, and do not span a full annual cycle; they therefore cannot capture seasonal or term-versus-vacation variation, and the estimates should be read as school-term, weekday-dominant rather than annual-average contact patterns. Larger surveys that deliberately sample weekends, holidays, and multiple seasons are needed to characterise temporal variation in contact patterns in Shangrao.

Measurement constraints also apply. Each respondent contributed only a single reported contact day, so within-person day-to-day variation could not be assessed, and contacts were self-reported for the previous day and are subject to recall and reporting bias, particularly for respondents with many contacts. For young children and for some older adults or visually impaired participants, the questionnaire was completed by a parent, family member, or community health worker, introducing possible proxy-reporting error.

## 5 Conclusion

Based on a survey of 2,101 respondents in Shangrao City, this study systematically characterized the social contact patterns of residents in this region. The mean daily number of contacts was 7.91, which is lower than previously reported estimates from first-tier cities such as Shanghai and Wuhan (6). The comparison with these studies suggests that the mean contact level observed in Shangrao was relatively low in the context of available Chinese contact surveys; however, this difference should be interpreted in light of variations in survey timing, sampling strategy, pandemic context, and contact definition.

The 5–19 years age group exhibited the highest contact frequency (9.99 individuals), and their household contacts were also significantly higher than those of other groups (4.88 individuals), underscoring the contribution of school congregation and household structure to overall contact intensity. This finding is consistent with evidence from other East Asian settings. A study conducted in South Korea demonstrated that the school-period contact matrix exhibits distinct clustering characteristics (8), and an empirical study from Japan revealed that social interactions display strong age assortativity, with approximately 40% of contacts occurring in schools or workplaces on weekdays (9). These convergent findings further indicate that during the early stages of respiratory infectious disease outbreaks, targeted and precise interventions in adolescent gathering settings—particularly schools—remain essential for interrupting transmission chains.

In addition to age, mobility range was a relatively robust factor associated with daily contact frequency. Respondents with a daily activity radius greater than 10 km reported more contacts, especially non-household contacts. This finding is consistent with mobility-based epidemiological studies suggesting that greater individual movement may increase opportunities for inter-regional mixing and facilitate the spatial spread of respiratory infectious diseases (10, 11), County-level differences were less stable and should be regarded as suggestive rather than definitive. These findings indicate that both age-specific contact patterns and population mobility should be considered in localized respiratory infectious disease modelling and prevention strategies.

Heat map analysis further revealed that social contact patterns in Shangrao City exhibited pronounced setting-specific clustering and age-dependent heterogeneity. The school environment represented the most densely populated space for social contact, particularly for the 5–19 years age group, whose mean daily contact frequency was higher than that of other groups, thereby identifying schools as priority settings for local infectious disease prevention and control. In addition, the age-specific contact matrix further demonstrated clear age-assortative mixing patterns, indicating that individuals tended to have more frequent contacts with people in similar age groups, particularly among school-aged populations. These findings emphasize the importance of incorporating local age-specific contact structures into respiratory infectious disease transmission models and early-warning analyses.

The findings of this study suggest that respiratory infectious disease surveillance and early-warning efforts in Shangrao City should prioritize high-contact age groups and settings, particularly adolescents, schools, and healthcare facilities. County-level differences, including those observed for Xinzhou District, should be interpreted as suggestive rather than definitive and require confirmation in future representative surveys. Through targeted interventions for adolescents and highly mobile populations, the transmission of pathogens from high-frequency social contact settings to households and communities may be more effectively reduced.

## Data Availability

The raw data supporting the conclusions of this article will be made available by the authors, without undue reservation.
