# Supplementary figures and images for: Social contact patterns and associated factors survey of Shangrao City
Source: Front Public Health. 2026 Jul 14;14:1866091. doi: 10.3389/fpubh.2026.1866091 (PMC13407773; doi:10.3389/fpubh.2026.1866091)

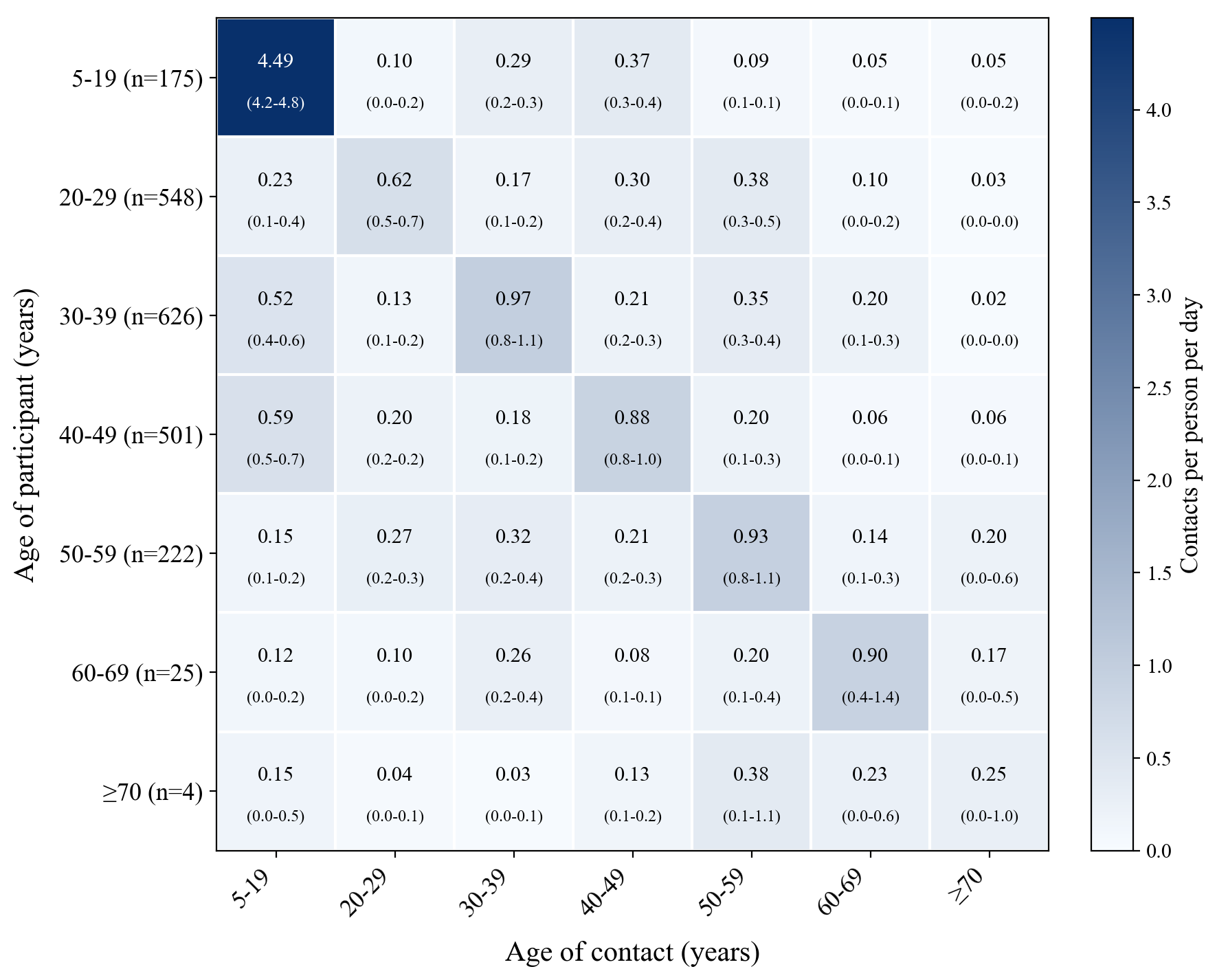

Supplement: Supplementary file 1 [file Image_1.PNG]

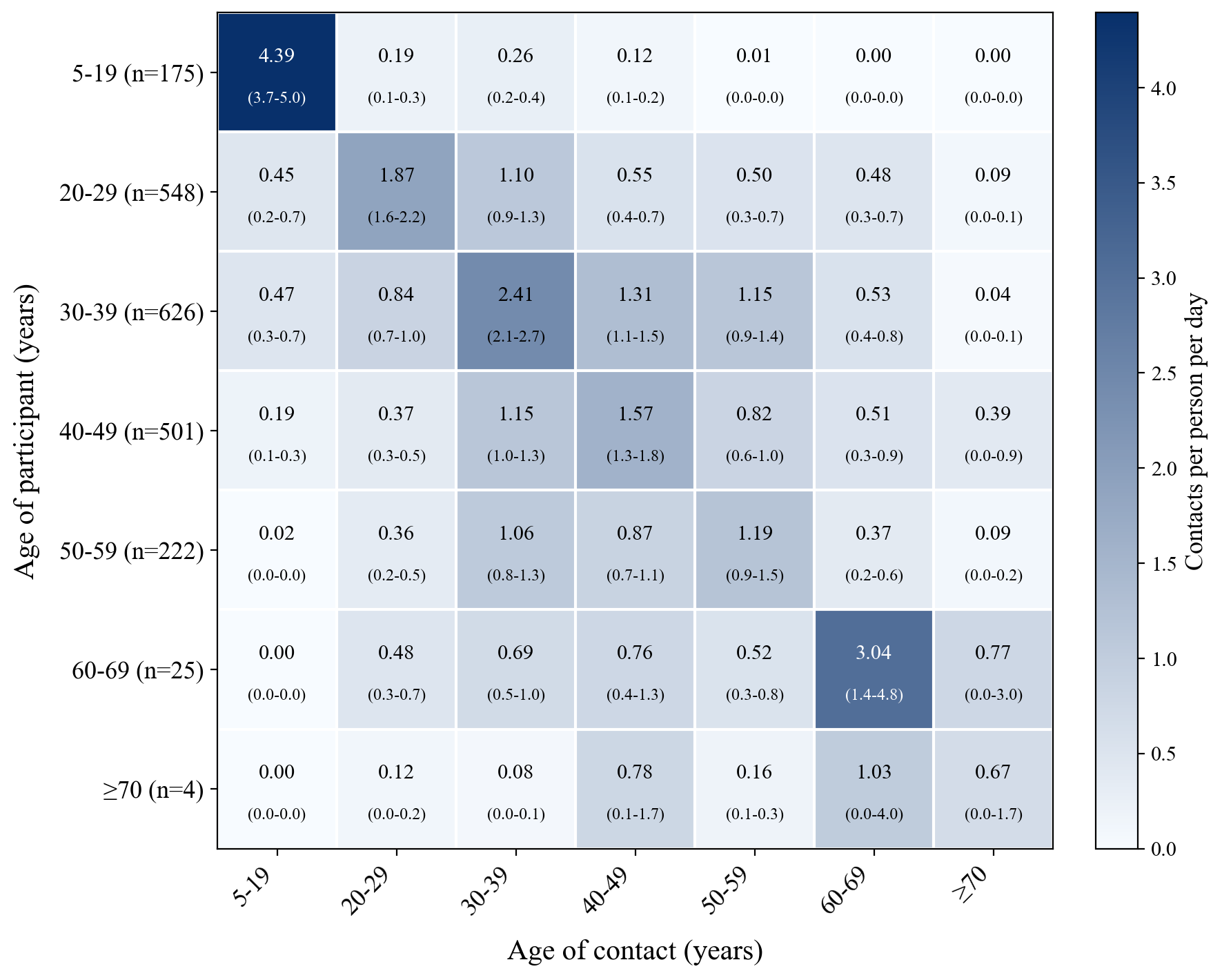

Supplement: Supplementary file 2 [file Image_2.PNG]
