# Supplementary material for: Social contact patterns and associated factors survey of Shangrao City
Source: Front Public Health. 2026 Jul 14;14:1866091. doi: 10.3389/fpubh.2026.1866091 (PMC13407773; doi:10.3389/fpubh.2026.1866091)
Supplement: Supplementary file 3 [file Supplementary_file_1.docx]

Supplementary File S1. Full Questionnaire

**Survey of Social Contact Patterns of Shangrao City Residents**

*(English translation of the original Chinese instrument)*

**Introduction shown to respondents on the landing page:**

*Dear participant, hello! We are the* ***Shangrao Center for Disease Control and Prevention*** *and the* ***Epidemiology Research Group of the School of Public Health, Xiamen University****. We are conducting research on contact patterns to learn how many people you contacted during the previous 24 hours. Your responses will be kept strictly confidential. Thank you for your assistance and support!*

**Participants are requested to report all individuals they contacted from 00:00 to 24:00 of the previous day.**

**Definition of contact:** A two-way conversation involving three or more words (e.g., “Hi!”, “Hello”, “How are you?”, “Well, how about you?”), or physical skin contact (e.g., handshake, hug, kiss, or other contact activity).

**Q1. Your gender:**

**☐ A.** Male

**☐ B.** Female

**Q2. Your age (years):**

(Drop-down menu options: <1, 1–4, 5–9, 10–14, 15–19, 20–24, 25–29, 30–34, 35–39, 40–44, 45–49, 50–54, 55–59, 60–64, 65–69, 70–74, ≥75)

**Q3. The area where you currently live is:**

**☐ A.** Urban area

**☐ B.** Rural area

**Q4. The district/county where you currently live:**

(Drop-down menu options: Xinzhou District, Guangxin District, Dexing City, Wuyuan County, Yushan County, Yanshan County, Hengfeng County, Yiyang County, Yugan County, Poyang County, Wannian County, Guangfeng District)

**Q5. Your range of activity yesterday:**

**☐ A.** Within 1 km (walking inside the neighborhood / school / village)

**☐ B.** 1–3 km (going out for groceries, not far)

**☐ C.** 4–10 km (working in a nearby area)

**☐ D.** More than 10 km (daily commute)

**Q6. Your educational attainment:**

**☐ A.** Primary school or below

**☐ B.** Junior high school

**☐ C.** Senior high / Vocational secondary school

**☐ D.** Junior college / Undergraduate

**☐ E.** Master’s degree or above

**Q7. Your usual place of study / work / daily life:**

(Drop-down menu options: School, Factory, Medical institution, Other)

**Q8. How many people do you live with?**

*(0 = living alone; 1 = one roommate or family member; and so on. Drop-down menu 0–20)*

**Q9. Information on co-residents:**

*(Fill in one row per co-resident; click “Continue filling” to add more rows.)*

| **Relationship to you** | **Number of people** | **Contact duration** | **Age** | **Contact mode** |
| --- | --- | --- | --- | --- |
|  |  |  |  |  |
|  |  |  |  |  |
|  |  |  |  |  |

**Options per column:**

Relationship: Family member / Classmate / Friend / Colleague / Other

Contact duration: < 5 min / 5–15 min / 15–60 min / 1–4 hours / > 4 hours

Age: <1, 1–4, 5–9, 10–14, 15–19, 20–24, 25–29, 30–34, 35–39, 40–44, 45–49, 50–54, 55–59, 60–64, 65–69, 70–74, ≥75

Contact mode: Physical contact / Non-physical contact

**Q10. How many people did you contact in total yesterday between 00:00 and 24:00 (excluding co-residents)?**

*(Drop-down menu options: 0, 1, 2, 3, 4, 5, 6, 7, 8, 9, 10, ≥11)*

**Q11. Information about the people you contacted:**

*(Fill in one row per person contacted; click “Continue filling” to add more rows.)*

| **Relationship to you** | **Number of people** | **Contact duration** | **Age** | **Contact mode** |
| --- | --- | --- | --- | --- |
|  |  |  |  |  |
|  |  |  |  |  |
|  |  |  |  |  |

*Options per column: identical to Q9.*

***End of questionnaire. Thank you for participating!***

**Notes on questionnaire administration**

• The questionnaire was administered online via the Wenjuanxing platform. Participants were recruited in communities, households, schools, and factories, and the questionnaire link or QR code was disseminated through the official WeChat account “Shangrao Disease Control” and work- and school-related WeChat groups.

• Yellow highlighting was applied to items where misinterpretation was considered likely (Q9, Q10, Q11) to draw respondents’ attention to the explanatory notes.

• For respondents who were unable to complete the survey independently (e.g., very young children, elderly persons with limited literacy or digital skills), the questionnaire was completed on their behalf by a parent, adult family member, or community health worker, who entered the respondent’s reported information on their behalf.

• Participation was voluntary; no monetary or material incentive was provided. Informed consent was obtained at the landing page of the questionnaire.
