# Supplementary material for: Social contact patterns and associated factors survey of Shangrao City
Source: Front Public Health. 2026 Jul 14;14:1866091. doi: 10.3389/fpubh.2026.1866091 (PMC13407773; doi:10.3389/fpubh.2026.1866091)
Supplement: Supplementary file 4 [file Table_1.docx]

Supplementary Table S1. Post-hoc multiple comparisons of total daily contacts across counties/districts (Tamhane’s T2 method) — full 12×12 matrix

|  | (I) District | (J) District | Mean Diff (I-J) | STD.Error | Sig. | 95% CI | |
| --- | --- | --- | --- | --- | --- | --- | --- |
|  |  |  |  |  |  | 95% CI lower | 95% CI Upper |
| Total contacts | Wuyuan | Dexing | -.849 | .360 | .708 | -2.06 | .36 |
|  |  | Wannian | -.980 | .422 | .749 | -2.41 | .45 |
|  |  | Xinzhou | -1.447* | .407 | .028 | -2.82 | -.07 |
|  |  | Guangxin | -.443 | .465 | 1.000 | -2.02 | 1.13 |
|  |  | Poyang | -1.248 | .473 | .444 | -2.86 | .36 |
|  |  | Yushan | -1.387 | .451 | .143 | -2.92 | .15 |
|  |  | Yiyang | -.835 | .515 | .999 | -2.59 | .92 |
|  |  | Guangfeng | -1.334 | .549 | .658 | -3.21 | .54 |
|  |  | Yanshan | -.395 | .492 | 1.000 | -2.08 | 1.29 |
|  |  | Hengfeng | .051 | .513 | 1.000 | -1.70 | 1.81 |
|  |  | Yugan | -.498 | .558 | 1.000 | -2.42 | 1.42 |
|  | Dexing | Wuyuan | .849 | .360 | .708 | -.36 | 2.06 |
|  |  | Wannian | -.131 | .427 | 1.000 | -1.58 | 1.32 |
|  |  | Xinzhou | -.597 | .412 | 1.000 | -1.99 | .80 |
|  |  | Guangxin | .406 | .469 | 1.000 | -1.19 | 2.00 |
|  |  | Poyang | -.399 | .478 | 1.000 | -2.02 | 1.23 |
|  |  | Yushan | -.538 | .456 | 1.000 | -2.09 | 1.01 |
|  |  | Yiyang | .014 | .520 | 1.000 | -1.76 | 1.79 |
|  |  | Guangfeng | -.484 | .553 | 1.000 | -2.38 | 1.41 |
|  |  | Yanshan | .454 | .496 | 1.000 | -1.24 | 2.15 |
|  |  | Hengfeng | .901 | .517 | .997 | -.87 | 2.67 |
|  |  | Yugan | .351 | .561 | 1.000 | -1.58 | 2.28 |
|  | Wannian | Wuyuan | .980 | .422 | .749 | -.45 | 2.41 |
|  |  | Dexing | .131 | .427 | 1.000 | -1.32 | 1.58 |
|  |  | Xinzhou | -.466 | .468 | 1.000 | -2.05 | 1.12 |
|  |  | Guangxin | .538 | .519 | 1.000 | -1.22 | 2.30 |
|  |  | Poyang | -.268 | .527 | 1.000 | -2.06 | 1.52 |
|  |  | Yushan | -.407 | .507 | 1.000 | -2.13 | 1.31 |
|  |  | Yiyang | .145 | .565 | 1.000 | -1.78 | 2.07 |
|  |  | Guangfeng | -.353 | .595 | 1.000 | -2.38 | 1.68 |
|  |  | Yanshan | .585 | .544 | 1.000 | -1.27 | 2.44 |
|  |  | Hengfeng | 1.032 | .563 | .990 | -.89 | 2.95 |
|  |  | Yugan | .482 | .603 | 1.000 | -1.58 | 2.55 |
|  | Xinzhou | Wuyuan | 1.447* | .407 | .028 | .07 | 2.82 |
|  |  | Dexing | .597 | .412 | 1.000 | -.80 | 1.99 |
|  |  | Wannian | .466 | .468 | 1.000 | -1.12 | 2.05 |
|  |  | Guangxin | 1.004 | .507 | .962 | -.71 | 2.72 |
|  |  | Poyang | .198 | .515 | 1.000 | -1.55 | 1.95 |
|  |  | Yushan | .059 | .495 | 1.000 | -1.62 | 1.74 |
|  |  | Yiyang | .611 | .554 | 1.000 | -1.27 | 2.50 |
|  |  | Guangfeng | .113 | .585 | 1.000 | -1.88 | 2.11 |
|  |  | Yanshan | 1.051 | .532 | .964 | -.76 | 2.86 |
|  |  | Hengfeng | 1.498 | .551 | .377 | -.38 | 3.38 |
|  |  | Yugan | .949 | .593 | 1.000 | -1.08 | 2.98 |
|  | Guangxin | Wuyuan | .443 | .465 | 1.000 | -1.13 | 2.02 |
|  |  | Dexing | -.406 | .469 | 1.000 | -2.00 | 1.19 |
|  |  | Wannian | -.538 | .519 | 1.000 | -2.30 | 1.22 |
|  |  | Xinzhou | -1.004 | .507 | .962 | -2.72 | .71 |
|  |  | Poyang | -.805 | .561 | 1.000 | -2.71 | 1.10 |
|  |  | Yushan | -.944 | .543 | .997 | -2.79 | .90 |
|  |  | Yiyang | -.392 | .597 | 1.000 | -2.42 | 1.64 |
|  |  | Guangfeng | -.891 | .626 | 1.000 | -3.02 | 1.24 |
|  |  | Yanshan | .048 | .577 | 1.000 | -1.92 | 2.01 |
|  |  | Hengfeng | .494 | .595 | 1.000 | -1.53 | 2.52 |
|  |  | Yugan | -.055 | .634 | 1.000 | -2.22 | 2.11 |
|  | Poyang | Wuyuan | 1.248 | .473 | .444 | -.36 | 2.86 |
|  |  | Dexing | .399 | .478 | 1.000 | -1.23 | 2.02 |
|  |  | Wannian | .268 | .527 | 1.000 | -1.52 | 2.06 |
|  |  | Xinzhou | -.198 | .515 | 1.000 | -1.95 | 1.55 |
|  |  | Guangxin | .805 | .561 | 1.000 | -1.10 | 2.71 |
|  |  | Yushan | -.139 | .550 | 1.000 | -2.01 | 1.73 |
|  |  | Yiyang | .413 | .604 | 1.000 | -1.64 | 2.47 |
|  |  | Guangfeng | -.086 | .633 | 1.000 | -2.24 | 2.07 |
|  |  | Yanshan | .853 | .584 | 1.000 | -1.14 | 2.84 |
|  |  | Hengfeng | 1.300 | .602 | .882 | -.75 | 3.35 |
|  |  | Yugan | .750 | .640 | 1.000 | -1.44 | 2.94 |
|  | Yushan | Wuyuan | 1.387 | .451 | .143 | -.15 | 2.92 |
|  |  | Dexing | .538 | .456 | 1.000 | -1.01 | 2.09 |
|  |  | Wannian | .407 | .507 | 1.000 | -1.31 | 2.13 |
|  |  | Xinzhou | -.059 | .495 | 1.000 | -1.74 | 1.62 |
|  |  | Guangxin | .944 | .543 | .997 | -.90 | 2.79 |
|  |  | Poyang | .139 | .550 | 1.000 | -1.73 | 2.01 |
|  |  | Yiyang | .552 | .587 | 1.000 | -1.44 | 2.55 |
|  |  | Guangfeng | .053 | .616 | 1.000 | -2.05 | 2.15 |
|  |  | Yanshan | .992 | .566 | .996 | -.94 | 2.92 |
|  |  | Hengfeng | 1.438 | .585 | .622 | -.55 | 3.43 |
|  |  | Yugan | .889 | .624 | 1.000 | -1.25 | 3.02 |
|  | Yiyang | Wuyuan | .835 | .515 | .999 | -.92 | 2.59 |
|  |  | Dexing | -.014 | .520 | 1.000 | -1.79 | 1.76 |
|  |  | Wannian | -.145 | .565 | 1.000 | -2.07 | 1.78 |
|  |  | Xinzhou | -.611 | .554 | 1.000 | -2.50 | 1.27 |
|  |  | Guangxin | .392 | .597 | 1.000 | -1.64 | 2.42 |
|  |  | Poyang | -.413 | .604 | 1.000 | -2.47 | 1.64 |
|  |  | Yushan | -.552 | .587 | 1.000 | -2.55 | 1.44 |
|  |  | Guangfeng | -.499 | .665 | 1.000 | -2.76 | 1.77 |
|  |  | Yanshan | .440 | .619 | 1.000 | -1.67 | 2.55 |
|  |  | Hengfeng | .886 | .635 | 1.000 | -1.28 | 3.05 |
|  |  | Yugan | .337 | .672 | 1.000 | -1.96 | 2.63 |
|  | Guangfeng | Wuyuan | 1.334 | .549 | .658 | -.54 | 3.21 |
|  |  | Dexing | .484 | .553 | 1.000 | -1.41 | 2.38 |
|  |  | Wannian | .353 | .595 | 1.000 | -1.68 | 2.38 |
|  |  | Xinzhou | -.113 | .585 | 1.000 | -2.11 | 1.88 |
|  |  | Guangxin | .891 | .626 | 1.000 | -1.24 | 3.02 |
|  |  | Poyang | .086 | .633 | 1.000 | -2.07 | 2.24 |
|  |  | Yushan | -.053 | .616 | 1.000 | -2.15 | 2.05 |
|  |  | Yiyang | .499 | .665 | 1.000 | -1.77 | 2.76 |
|  |  | Yanshan | .939 | .647 | 1.000 | -1.27 | 3.14 |
|  |  | Hengfeng | 1.385 | .663 | .921 | -.87 | 3.65 |
|  |  | Yugan | .836 | .698 | 1.000 | -1.55 | 3.22 |
|  | Yanshan | Wuyuan | .395 | .492 | 1.000 | -1.29 | 2.08 |
|  |  | Dexing | -.454 | .496 | 1.000 | -2.15 | 1.24 |
|  |  | Wannian | -.585 | .544 | 1.000 | -2.44 | 1.27 |
|  |  | Xinzhou | -1.051 | .532 | .964 | -2.86 | .76 |
|  |  | Guangxin | -.048 | .577 | 1.000 | -2.01 | 1.92 |
|  |  | Poyang | -.853 | .584 | 1.000 | -2.84 | 1.14 |
|  |  | Yushan | -.992 | .566 | .996 | -2.92 | .94 |
|  |  | Yiyang | -.440 | .619 | 1.000 | -2.55 | 1.67 |
|  |  | Guangfeng | -.939 | .647 | 1.000 | -3.14 | 1.27 |
|  |  | Hengfeng | .447 | .616 | 1.000 | -1.66 | 2.55 |
|  |  | Yugan | -.103 | .654 | 1.000 | -2.34 | 2.13 |
|  | Hengfeng | Wuyuan | -.051 | .513 | 1.000 | -1.81 | 1.70 |
|  |  | Dexing | -.901 | .517 | .997 | -2.67 | .87 |
|  |  | Wannian | -1.032 | .563 | .990 | -2.95 | .89 |
|  |  | Xinzhou | -1.498 | .551 | .377 | -3.38 | .38 |
|  |  | Guangxin | -.494 | .595 | 1.000 | -2.52 | 1.53 |
|  |  | Poyang | -1.300 | .602 | .882 | -3.35 | .75 |
|  |  | Yushan | -1.438 | .585 | .622 | -3.43 | .55 |
|  |  | Yiyang | -.886 | .635 | 1.000 | -3.05 | 1.28 |
|  |  | Guangfeng | -1.385 | .663 | .921 | -3.65 | .87 |
|  |  | Yanshan | -.447 | .616 | 1.000 | -2.55 | 1.66 |
|  |  | Yugan | -.549 | .670 | 1.000 | -2.84 | 1.74 |
|  | Yugan | Wuyuan | .498 | .558 | 1.000 | -1.42 | 2.42 |
|  |  | Dexing | -.351 | .561 | 1.000 | -2.28 | 1.58 |
|  |  | Wannian | -.482 | .603 | 1.000 | -2.55 | 1.58 |
|  |  | Xinzhou | -.949 | .593 | 1.000 | -2.98 | 1.08 |
|  |  | Guangxin | .055 | .634 | 1.000 | -2.11 | 2.22 |
|  |  | Poyang | -.750 | .640 | 1.000 | -2.94 | 1.44 |
|  |  | Yushan | -.889 | .624 | 1.000 | -3.02 | 1.25 |
|  |  | Yiyang | -.337 | .672 | 1.000 | -2.63 | 1.96 |
|  |  | Guangfeng | -.836 | .698 | 1.000 | -3.22 | 1.55 |
|  |  | Yanshan | .103 | .654 | 1.000 | -2.13 | 2.34 |
|  |  | Hengfeng | .549 | .670 | 1.000 | -1.74 | 2.84 |
| *. The significance level of the mean difference is 0.05. | | | | | | | |
